# REASSURED Multiplex Diagnostics: A Critical Review and Forecast

**DOI:** 10.3390/bios12020124

**Published:** 2022-02-16

**Authors:** Jonas A. Otoo, Travis S. Schlappi

**Affiliations:** Keck Graduate Institute, Claremont, CA 91711, USA; jotoo18@students.kgi.edu

**Keywords:** diagnostics, multiplex, point-of-care diagnostics, REASSURED

## Abstract

The diagnosis of infectious diseases is ineffective when the diagnostic test does not meet one or more of the necessary standards of affordability, accessibility, and accuracy. The World Health Organization further clarifies these standards with a set of criteria that has the acronym ASSURED (Affordable, Sensitive, Specific, User-friendly, Rapid and robust, Equipment-free and Deliverable to end-users). The advancement of the digital age has led to a revision of the ASSURED criteria to REASSURED: Real-time connectivity, Ease of specimen collection, Affordable, Sensitive, Specific, User-friendly, Rapid and robust, Equipment-free or simple, and Deliverable to end-users. Many diagnostic tests have been developed that aim to satisfy the REASSURED criteria; however, most of them only detect a single target. With the progression of syndromic infections, coinfections and the current antimicrobial resistance challenges, the need for multiplexed diagnostics is now more important than ever. This review summarizes current diagnostic technologies for multiplexed detection and forecasts which methods have promise for detecting multiple targets and meeting all REASSURED criteria.

## 1. Introduction

Clinical diagnostics are devices or methods that are used to detect biomarkers in the genome, proteome and metabolome for diagnosis, subclassification, prognosis, susceptibility risk assessment, treatment selection, and response to therapy monitoring [1,2]. Biomarker analytes include nucleic acids, proteins, peptides, lipids, metabolites, and other small molecules [3,4]. Diagnostic tests are generally carried out in central labs, clinics, hospitals, doctors’ offices, and point-of-care (POC) settings. Thousands of diagnostic tests have been developed over the years, with varying levels of complexity, turnaround time, cost, and other factors. While diagnostics account for less than 5% of hospital costs and ~1.6% of all Medicare costs, they influence up to 60–70% of healthcare decision making [5]. There are several stakeholders in diagnostics, each with their own priorities: patients, healthcare providers, payers, pharmaceutical companies, diagnostic device manufacturers, local and international health organizations, governments, public health agencies, and regulatory bodies [6,7].

In order to be FDA (Food and Drug Administration) approved, diagnostic tests need to meet certain standards for analytical and clinical validation. Analytical validation assesses the sensitivity, specificity, accuracy, and precision of the test. Clinical validation assesses the ability of the test to achieve its intended aim. Diagnostic tests in hospitals or reference labs are able to meet analytical and clinical standards for accuracy and performance because complexity and cost are not an issue. It is much more difficult for point-of-care diagnostics, however, which must also minimize cost and complexity in their design and manufacturing. The World Health Organization Special Program for Research and Training in Tropical Diseases (WHO/TDR) concluded, in a study in 2003, that POC diagnostics should meet the ASSURED (Affordable, Sensitive, Specific, User-Friendly, Rapid, Equipment-Free, Delivered) criteria [8]. In 2006, the WHO/TDR further recommended the ASSURED criteria as a benchmark to decide whether diagnostic tests address disease control needs [9]. The ASSURED criteria represent three main attributes that are significant for a diagnostic test. These attributes are accessibility, affordability, and accuracy. While all three attributes are important, it is very challenging for any diagnostic test to adequately possess all three. The different stakeholders in diagnostics may have varying orders of priority among the three attributes. Patients may want diagnostic tests that are first, affordable, second, accessible, and third, accurate. Healthcare providers likely prefer accuracy, accessibility, and then affordability. Governments may prioritize accessibility over affordability and accuracy. Manufacturers of diagnostics that are maximizing profits probably emphasize accessibility, accuracy, and then affordability.

In the face of the SARS-CoV-2 pandemic, the role and importance of diagnostics has become increasingly apparent. Diagnostics help to track, contain, and control the spread of infectious diseases. Several diagnostic tests were developed in the wake of the SARS-CoV-2 pandemic [10,11,12,13], which guided the formulation and implementation of measures that were used to protect the public, find new variants, track the disease, and slow its spread. Diagnostics have also played a major role in non-infectious diseases. Early detection of biomarkers of cancer, cardiovascular disease and metabolic diseases, such as diabetes and hypertension, have reduced the mortality rate of humans over the years [14,15,16,17].

### 1.1. Multiplexed Diagnostics

Multiplexing is the process of simultaneously detecting or identifying multiple biomarkers in a single diagnostic test, which can be valuable for several different types of diseases. For example, pharmacogenomic studies in patients with cardiovascular disease have indicated that the presence of polymorphisms affects patients’ response to various drugs [18]. Therefore, the multiplex detection of relevant biomarkers will not only provide insight of the pathophysiology of cardiovascular disease, but also provide a guide for the most efficient treatment option. Most cancers have biomarkers in common with other cancers, hence detecting multiple biomarkers is needed for the accurate differentiation of cancer types or location [19,20]. Hermann et al. [21] demonstrated that several biomarkers are significantly elevated in breast cancer patients versus patients with benign breast tumor disease. The multiplexed detection of these biomarkers enables oncologists to accurately diagnose their patients and select the appropriate therapy, thus improving patient outcomes and decreasing healthcare costs. Cytokines are important in the mediation of immune responses, such inflammation and mobilization of immune cells [22]. They are secreted by different cell types and are very diverse [23]. Multiplexed detection of cytokines is key to the better understanding of the immune response. Abdullah et al. [24] demonstrated that multiplexed detection of cytokines was important to understand whether neural stem cell rosette morphologies had an impact on the profile of cytokine signals and therefore had different outcomes in neurodegenerative disease cell therapies.

**Figure 1:**
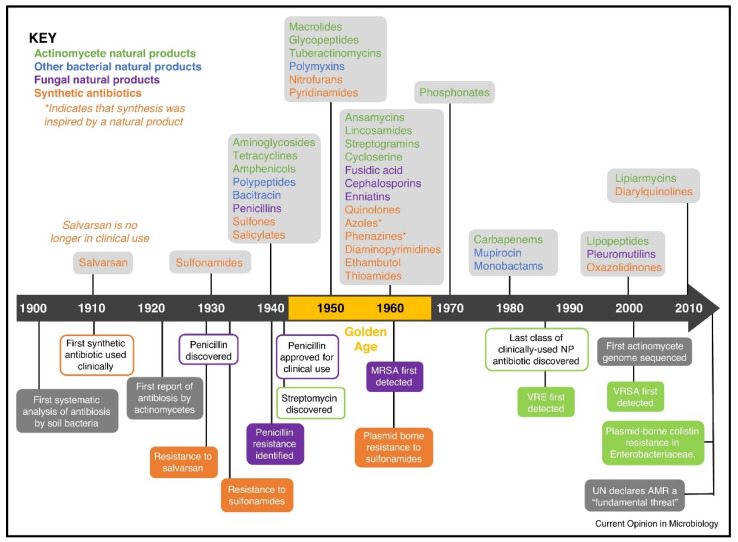
The timeline of antibiotics class discovery/development and onset of antimicrobial resistance reproduced from [29].

**Figure 2:**
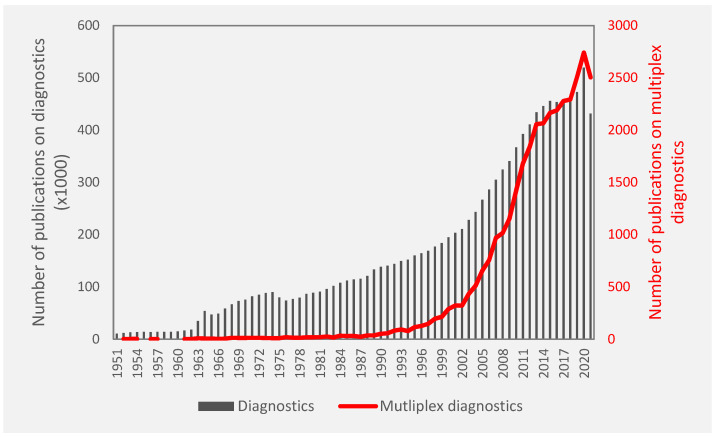
Annual publications related to diagnostics compared to annual publications related to multiplex diagnostics from 1950 to 2021 from the PubMed database (National Center for Biotechnology Information).

Infectious disease is another area where multiplexed diagnostics are extremely valuable. Most infectious diseases, such as urinary tract infections and respiratory infections, have multiple causative pathogens, but the resulting symptoms do not indicate the causative pathogen. On the other hand, different types of infections that have shared symptoms could be misdiagnosed or incompletely diagnosed. For example, SARS-CoV-2 and influenza A or B present with many of the same symptoms and clinical features [25,26]. Studies show that there is the prevalence of influenza coinfection among people with SARS-CoV-2 is 0.4% in the United States of America and 4.5% in Asia [27]. In a case study of 1986 patients that presented with Severe Acute Respiratory Infection (SARI), 14.3%, 8.8% and 0.3% had SARS-CoV-2, influenza and SARS-CoV-2/influenza coinfection, respectively [28]. In another study, 40% of a cohort of Kenyans who sought treatment for fever were presumed to have malaria and received malaria medicines even though they actually had HIV [29]. Incomplete diagnosis of infectious disease leads to inefficient treatments by exposing some pathogens to sub-lethal doses or the wrong antibiotics. This contributes to the emergence of antimicrobial resistance and recurrent infections as well as persistent secondary infections [30,31]. The last two classes of antibiotics were discovered in 1987 and 2004 [32], and since then, we are in a period of discovery void while there is rapid emergence of antimicrobial pathogens to the antibiotics that currently exist (Figure 1). According to O’Neil [33], 10 million people will die annually due to antimicrobial resistance (AMR) by 2050. Furthermore, AMR-related costs and the associated loss of productivity amount to about USD 55 billion annually in the U.S. alone [34]. Better diagnostics and treatment for tuberculosis could save 770,000 lives over the course of 2015 to 2025 [33], while a malaria test could save ~2.2 million lives and prevent ~447 million unnecessary treatments per year [35]. The introduction of antibiotics increased the average lifespan of humankind by 23 years since the first introduction of antibiotics, thus showing the drastic consequences if we were to lose the use of antibiotics that we currently have [32]. Another instance where multiplexing is crucial is the diagnosis of blood infections. Sepsis resulting from blood infections can be caused by many pathogens and becomes increasingly fatal over time, with mortality increasing by 7.6% for every hour that passes without receiving the correct antibiotic [36]. Accurately identifying which pathogen(s) is responsible for the blood infection is therefore a race against time to start the antibiotic therapy before sepsis becomes fatal [37]. The diagnosis of infections should therefore be approached by syndromic diagnosis, wherein all the potential pathogens for an infection or symptom are investigated rather than tested for just the most likely pathogen and then conducting other tests if negative [38,39]. Multiplexed diagnostic tests—wherein one sample is simultaneously tested for multiple pathogens in the same device—are essential for blood infections nowadays and important to combat AMR for all types of infections in the future. A query on the PubMed database of the National Center for Biotechnology Information (NCBI) suggests that researchers have become increasingly more interested in multiplex diagnostics (Figure 2).

### 1.2. REASSURED Diagnostics

Considering the advances in digital technology and mobile health, a new REASSURED (Real-time connectivity, Ease of specimen collection, Affordable, Sensitive, Specific, User-Friendly, Rapid and Robust, Equipment free or simple Environmentally friendly, Deliverable to end-users) framework has been proposed as the benchmark for diagnostic systems [40]. The diagnosis of a disease is just the first step. The information from the diagnosis results needs to be used to inform actionable steps to treat or manage the disease. In a remote setting where a healthcare professional is not readily accessible, real-time connectivity provides the avenue to transmit the results to the healthcare professional for medical advice. Furthermore, having a reader that can provide the results of a diagnostic test is important especially in ambiguous cases where there is uncertainty due to variation in the interpretation of the results. A reader will serve as a standardized way to state the results of the diagnostic test [41,42,43].

The development of diagnostic tests that meet all the ASSURED criteria, but uses hard-to-obtain samples, such as venous blood, will not be very helpful in the absence of a trained professional to obtain the sample. It is therefore very crucial that, when possible, diagnostic tests should be developed to use easy-to-obtain and non-invasive samples, such as finger pricks, nasal or oral swabs, or urine samples.

While all the elements of the REASSURED criteria are important for POC diagnostics, it is challenging for any diagnostic device to embody all of these elements and trade-offs are often made in one or more elements to achieve other elements. For instance, nucleic acid testing (NAT) is very sensitive and specific, but often requires purification or isolation of the nucleic acid, concentration of the nucleic acid, amplification, and detection of the nucleic acid [44,45,46]. These processes can be achieved through user steps or by the introduction of equipment components that can execute them. On the other hand, antigen-based diagnostics, such as a lateral flow assay, are not as sensitive and specific as NAT, but are far more user-friendly, affordable, rapid, and deliverable [47]. In these two scenarios, some degree of sensitivity and specificity could be traded for the affordability, user-friendliness, and equipment complexity of the diagnostic test by detecting antigens instead of nucleic acids.

Naseri et al. [48] have summarized POC devices based on lateral flow assays (LFAs) and paper-based analytical devices (PADS) technology that were developed in the last 10 years for common human viral infection diagnostics. Dincer et al. [49] presented a survey of the existing multiplexed POC tests in academia and industry, while Kim et al. [50] summarized current POC tests for multiplex molecular testing of syndromic infections; however, these reviews focused mainly on POC diagnostics rather than summarizing devices that meet REASSURED criteria. In this paper, we present the current state of multiplexed diagnostic technology that meet REASSURED criteria based on an in-house developed scoring scheme. This review summarizes multiplexed diagnostics in three categories: (i) clinically used, (ii) in academia or research only, and (iii) next-generation technology. We then discuss the limitations in developing multiplexed REASSURED diagnostics, present current gaps in technology, and describe the needs for future research and development. For the purpose of this review, clinical diagnostics refer to diagnostics that have been approved by the FDA (including Emergency Use Authorization) or have a CE marking and are available for patient diagnosis.

## 2. Clinically Available Multiplexed Diagnostics

### 2.1. Proteins and Peptides

Multiplex detection of select protein or peptide biomarkers in human samples, such as blood, serum, saliva and urine for clinical diagnosis, while very important, presents with a challenging puzzle: human samples typically have a myriad of diverse proteins and peptides [51], only some of which are the protein of interest. Accurately differentiating the select protein biomarkers out of the matrix is challenging due the occurrence of cross-reactivity [52]. The advancement in technology has made it possible for some immunoassays to be adapted to the point-of-care setting for multiplex peptide and protein biomarker detection. LFAs use a variety of detection techniques, such as fluorescent immunoassays (FIA), chemiluminescence immunoassay [53] and colorimetric immune assays [54], for the detection of protein and peptide biomarkers. While LFAs have lower sensitivity compared to molecular diagnostic tests [55], they are rapid and relatively cheaper to fabricate compared to other diagnostics [56]. LFAs were the first tests that meet the WHO ASSURED criteria [47,57]. They are typically equipment free or are accompanied by a simple reader with a digital interface. When immunoassays, such as LFAs, have a colorimetric read-out, the interpretation of the results is subjective to the person who is reading the results. This may be problematic in cases where the biomarkers being detected are present in low concentrations. Utilizing a simple reader in conjunction with these LFAs will promote an objective and a more accurate interpretation of the results. This will also enable the LFAs to satisfy the REASSURED criteria.

Enzyme-Linked Immunosorbent Assays (ELISAs) are a highly sensitive method for the detection of protein and peptide biomarkers. ELISAs are very prone to interferences [58], which pose challenges to developing a multiplex test. This challenge is overcome through the use of spatial multiplexing approaches, such as wells and microarrays [59,60]. To avoid false positive tests as a result of non-specific interactions, there are multiple wash steps in ELISA assays. The automation of ELISAs for adaption to the POC and limited resource settings is therefore challenging because complex equipment components are required for fluid handling to execute wash steps. Furthermore, to avoid false negative tests, there are lengthy incubation periods in ELISA assays. It is therefore very challenging to adapt ELISAs for point-of-care diagnostics that fit the REASSURED criteria.

The BinaxNOW influenza A and B card 2 developed by Abbott is a multiplex immunochromatographic LFA that is able to provide rapid differential diagnosis of influenzas A and B infection [61]. This test is designed to be read by the DIGIVAL reader for result interpretation. The DIGIVAL reader is portable and battery powered, making it suitable for limited resource settings. Becton and Dickinson’s (BD) Veritor™ Flu A + B with analyzer distinguishes between influenzas A and B as well. The BD test analyzer is palm sized and battery powered and hence suitable for use at remote and limited resource settings [62]. Acucy influenza A and B test developed by Sekisui diagnostics comes with a portable battery-powered reader as well [63]. Quidel’s Sofia 2 Flu + SARS antigen FIA test is a multiplex fluorescent immunoassay for the detection of and differentiating SARS-CoV-2, influenzas A and B [64]. The Sofia 2 reader is portable, but it is not battery powered. It is suitable for point-of-care settings, but it may not be fitting for a remote or limited resource setting. There appears to be a trend of LFA diagnostics being accompanied by readers and real-time connectivity [41,42,43], hence rapidly adapting and meeting the REASSURED criteria.

### 2.2. Nucleic Acids

Polymerase chain reaction (PCR) is the gold standard amplification method for molecular diagnostic assays for clinical use. PCR-based diagnostics assays are robust and can use crude samples, such as blood [65]. The key obstacle preventing PCR NATs from meeting all of the ASSURED criteria is that multiple temperatures are required for the amplification of target NAs. Device components that can perform thermal cycling are therefore necessary when developing a PCR-based diagnostic device. It is also challenging to develop multiplex PCR diagnostics. The existence of multiple primers for multiple targets increases the rate of formation of primer dimers, which then leads to non-specific amplification [66]. There is therefore a need for the stringent optimization of reaction conditions and parameters in order to achieve a multiplex PCR [67]. On the other hand, isothermal amplification, such as loop-mediated isothermal amplification (LAMP) and recombinase polymerase amplification (RPA), do not require thermal cycling [68,69]. The sensitivity of LAMP is not affected when the nucleic acid sample is impure and has other crude components, such as proteins and other cellular components [70]. However, a LAMP reaction requires four to six primers for each target, and hence poses a challenge when multiplexing due the occurrence of non-specific amplification [69,71].

The Accula dock developed by Mesa Biotech (now a part of Thermo Fisher Scientific (Waltham, MA, USA.)) is a portable sample-to-answer molecular diagnostic device that uses Mesa Biotech’s proprietary PCR technology OSCillating amplification reaction (OSCAR) [72]. The Accula systems operates with a test cassette in which the multiplexed nucleic acid detection occurs. The Accula Flu A and Flu B is CLIA waived the multiplexed test for the detection of influenzas A and B, and the device has a 510K FDA clearance [73]. The disposable test cassette together with the dock are a portable system that checks nearly all the criteria for REASSURED diagnostics.

**Figure 3:**
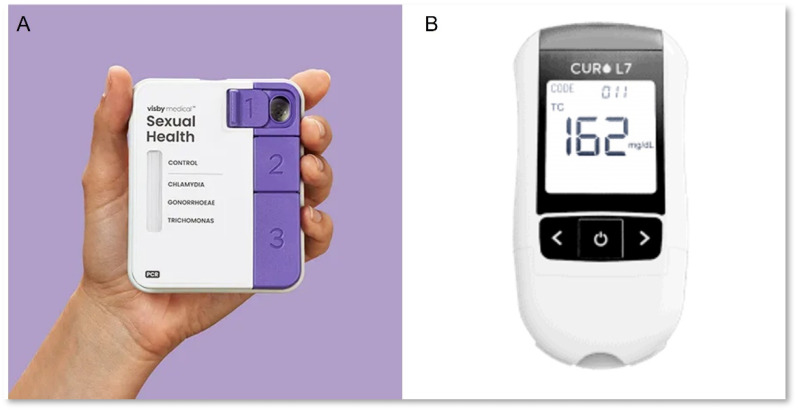
Clinical diagnostics devices scored on the REASSURED scoring scheme: (**A**) Visby Medical Sexual Health reproduced with permission from Visby Medical (https://www.visbymedical.com/resources/press-kit/ (accessed on 10 February 2022)). (**B**). Curo L7 reproduced with permission from Curofit (https://curofit.com/ (accessed on 10 February 2022)).

The Visby Medical Sexual Health (Figure 3A) developed by Visby Medical is a handheld device that is capable of a rapid multiplexed PCR for the detection of *Chlamydia trachomatis*, *Neisseria gonorrhoeae*, and *Trichomonas vaginalis* [74]. The Visby Medical Sexual Health device recently received CLIA waiver and FDA clearance. The device is a disposable sample-to-answer diagnostic, which makes it adaptable for point-of-care testing and in remote settings. Visby medical’s diagnostic device can be adaptable to any form of multiplexed molecular diagnostic test, as the Visby Medical COVID-19 test has been granted Emergency Use Authorization (EUA) by the FDA for use by authorized labs [75].

Biomeme’s Franklin three9 is a rechargeable battery-operated mobile thermocycler that is capable of conducting a multiplexed detection of nucleic acids and is adaptable to limited resource settings. It is capable of PCR, (Reverse Transcriptase) RT-PCR, (quantitative) qPCR and isothermal amplification. Franklin is not a sample-to-answer platform as it requires upstream steps sample preparation. However, the sample preparation steps can be achieved in about 1–2 min using Biomeme’s M1 sample-prep cartridge kits. The Franklin system has Bluetooth and a wireless connection capability and is accompanied by an intuitive companion mobile app that facilitates wireless programing and managing of experiments. The Franklin three9 is capable of simultaneously testing nine samples with three targets each [76].

### 2.3. Small Molecules, Lipids, and Other Biomarkers

CardioChek PA Analyzer by PTS Diagnostics is a portable handheld diagnostic device that is battery operated. It works in conjunction with panels test strips to measure single and multiplex analytes. The CardioChek PA analyzer and test strips can measure total cholesterol, high density lipoproteins, triglycerides and glucose and provide results in 45 to 90 s. The test strips are stable at room temperature [77].

Curofit’s Curo L7 m (Figure 3B) is capable of multiplex runs with up to six simultaneous tests with a cholesterol test strip. The device is handheld and battery-powered and is able to deliver results directly from sample. The Curo L7 m is suitable for point-of-care and low resource settings [78].

## 3. Multiplexed Diagnostics in Research or Academia

### 3.1. Proteins and Peptides

**Figure 4:**
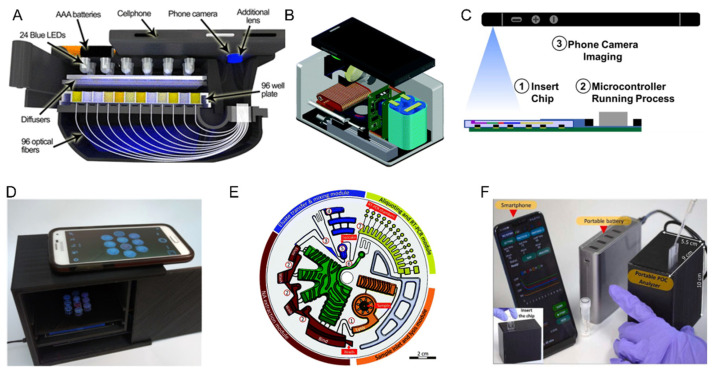
Multiplex diagnostics systems in *research and academia*. (**A**) Smartphone ELISA plate reader system reproduced from [56]. (**B**) FGAS system reproduced from [76] with permission from the Royal Society of Chemistry. (**C**) Smartphone-based ELISA-on-a-chip reproduced from [74], with the permission of AIP Publishing. (**D**) MELISA platform reproduced from [57] with permission from the Biosensors and Bioelectronics. (**E**) RespiDisk system reproduced from [77]. (**F**) IoT-based diagnostic system reproduced [78] with permission from the Biosensors and Bioelectronics.

There are many multiplex immunoassays (MIAs) under development and only a few have been commercialized [79]. Chen et al. [80] demonstrated the use of a smartphone camera for reading ELISA-on-a-chip assays (Figure 4C). Berg et al. [59] published a cellphone-based hand-held microplate reader (Figure 4A) that used optical fibers to transmit data from ELISA plates to a cell-phone camera for diagnostics at the point of care. Mobile-phone-based ELISA (MELISA) is a portable system published by Zhdanov et al. [60] (Figure 4D). It is a miniature version of ELISA that is capable of executing all ELISA steps as well as providing a phone-based read-out of the results. The MELISA system has multiple reaction wells and has the potential to developed into a multiplexed system. According to the publishers, the total assembly of the MELISA system cost about USD 35. The system does not require any complex instrumentation; however, it uses plasma and hence requires an upstream sample preparation step. Ghosh et al. [81] described a microchannel capillary flow assay that detected malaria by a smartphone-assisted chemiluminescence-based ELISA. Perhaps, mobile phone-based ELISA platforms are the future direction for REASSURED diagnostics for protein and peptide biomarker detection.

### 3.2. Nucleic Acids

Shu et al. [82] proposed rapid multiplexed molecular diagnostic system dubbed flow genetic analysis system (FGAS) that is capable of conducting quantitative detection of nucleic acids (Figure 4B). FGAS is portable and battery powered, making it suitable for low resource settings. It is coupled with a smartphone, which is used for fluorescent imaging. RespiDisk (Figure 4E) is a fully automated multiplex molecular diagnostic device for respiratory tract infections [83]. The platform is based on RT-PCR and capable of automated sample-to-answer analysis, with a turnaround time of 3 h and 20 min. The RespiDisk system operates by centrifugal microfluidics. An Internet of things (IoT)-based diagnostic device is presented by Nguyen et al. [84] (Figure 4F). This platform is accompanied by an integrated microfluidic chip that is capable of running a multiplexed reverse-transcriptase LAMP (RT-LAMP) reaction. In addition, this battery-powered portable device has optical detection capability and was able to accurately detect SARS-CoV-2 from clinical samples in 33 min. The advanced IoT based device can be operated with a smartphone and provides real-time data to the user. It is capable of sample-to-answer analysis and hence there are only few user steps. Carter et al. [85] presented a multiplex lateral flow microarray platform for the detection nucleic acids. This platform combined the desirable qualities of an isothermal nucleic acid test (high sensitivity, high specificity, and no thermal cycling) with the best qualities LFAs (inexpensive, rapid, and equipment-free).

## 4. Next Generation Multiplex Diagnostics

The development of microfluidics and nanofluidics has inspired the emergence of several miniaturized platforms, such as lab-on-a-chip and lab-on-a-disk. These platforms present the capabilities of molecular-scale sensitivity on low-cost and rapidly fabricated devices [86,87,88]. However, the adoption of these platforms into clinical diagnostics are yet to be realized. Yeh et al. [89] presented a microfluidic chip called SIMPLE (Self-powered Integrated Microfluidic Point-of-care Low-cost Enabling). The SIMPLE chip is portable and completely integrated, allowing the accurate quantitative detection of nucleic acids from whole blood in 30 min. The emergence of microfluidic technologies propelled the development of digital PCR (dPCR). dPCR offers advantages, such as excellent precision [90], single copy detection, high sensitivity and absolute quantification [91]. Droplet microfluidics [92,93,94] and microarray [95,96] are some of the techniques used to achieve multiplexing by dPCR. While not able to meet all REASSURED criteria, some dPCR techniques show potential by using a mobile phone for detection and using simple fluid handling methods [97,98]. While very promising, the development and commercialization of microfluidic platforms are hindered by setbacks, such as the high cost and complexity of manufacturing on large scale, and challenges of integration from sample to answer [99,100].

In recent years, a number of studies are migrating towards the application of CRISPR/Cas systems for multiplex molecular diagnostics [101,102,103,104]. Gootenberg et al. [102] presents SHERLOCKv2, a multiplex platform for nucleic acid detection with high sensitivity and specificity and is integrated with a lateral flow read out. This presents the potential for SHERLOCKv2 to be developed into a multiplex and portable platform for diagnostics. Recently, Ackerman et al. [105] proposed a high throughput multiplex nucleic acid detection microarray system called CARMEN-Cas13. The high sensitivity and specificity of CARMEN combined with its incredibly high throughput, endows it with the potential of being the ultimate point of care diagnostic device when integrated with upstream sample preparation and concentration steps. Rezaei et al. [106] recently developed a portable device for the screening of SARS-CoV-2 by RT-LAMP and followed by CRISPR/Cas12a reaction and FAM-biotin system to give a fluorescent readout in a LFA. The device is semiautomated and battery operated. It has the potential for multiplexing and is able to produce results in about an hour. Yi et al. presented a similar system termed CRICOLAP for the detection of SARS-CoV-2 and also employs an amplification step by RT-LAMP, which is followed by a CRISPR/Cas12a collateral cleavage system for target recognition [107]. The paper reports a real-time parallel fluorescent readout system.

In the current digital age, next-generation diagnostics are combined with machine learning capabilities for high throughput and highly accurate results. Ballard et al. [108] demonstrated a multiplexed paper-based Vertical Flow Assay (VFA) platform that used a deep learning-based framework for sensing and quantifying high sensitivity C-Reactive Protein. This platform represents a low-cost device that can be adapted for molecular diagnostics at the POC and low resource settings. Machine-learning-assisted dPCR has also improved diagnostic outcomes as demonstrated by Liu [109] and Miglietta [110].

## 5. Discussion

In the REASSURED scoring scheme (Table 1), LFAs with an in-built or a combined reader had low sensitivity and specificity scores compared to molecular diagnostics, but they had high overall scores. LFAs have been widely adopted for rapid diagnostics for decades and while they are more affordable and simpler to develop and/or use, they do not have good sensitivity and have low multiplex capacity. Most LFAs can only multiplex two or three types of biomarkers. The limitations to multiplexing capability of LFAs are due to technical and operational challenges, such cross-reactivity and selection of appropriate diluents [56,111]. Most proteins or peptides have unique charges and pH and hence, unique isoelectric points in different buffer conditions. There is therefore a challenge of selecting the appropriate buffer for the select protein and peptide biomarkers to multiplexed. In infectious diseases, acquired immune responses do not occur until several days after exposure, and the antibodies linger in the body for days after the pathogen has been cleared [112]. This makes it difficult for LFAs to distinguish between an active and inactive infection.

The reviewed molecular diagnostics demonstrated much higher multiplex capacity compared to the LFAs. Molecular diagnostics are easier to multiplex than LFAs because biomarker recognition is achieved through the highly specific complementary hybridization of primers and/or probes. The quest to bring molecular diagnostic devices to the point-of-care setting has led to an increased focus on the miniaturization of the test systems. A major challenge that is often encountered by the miniaturization of the molecular diagnostic test platforms is the integration of sample preparation steps. Sample preparation include steps for isolation, purification, and concentration of nucleic acids from crude samples, such as blood and saliva. While the execution of these steps increases the sensitivity and specificity of molecular diagnostics, they are a major driver in the cost and complexity of these devices. Molecular diagnostics that have in-built readers or connectivity to smartphones were completely integrated from sample to answer, and handheld and battery-powered devices generally scored the highest points on the multiplexed REASSURED scoring scheme.

There is a need for technology that is highly accurate, but also is affordable and accessible, especially in the developing world. Such a technology will not only help to address the need for increased access to diagnostics, but also ensure endemic and pandemic preparedness for the future. More funds need to be allocated to the development of multiplexed REASSURED diagnostics through funding by research and academic institutions and the incentivizing of research and development efforts of industry.

Point-of-care diagnostics development should gravitate towards more syndromic test panels, such as respiratory infection panels, urinary tract infection panels, blood protein panel and STI panels. Multiplexed panel measurements rather than single panel measurements are important because they facilitate the efficient and effective diagnosis of syndromic infections, accurately indicate the correct antibiotic or treatment, and minimize the number of tests that need to be run to diagnose coinfections.

Novel technologies in development that meet the REASSURED criteria should be incentivized by governments and international organizations to bring them to the market. Gene Xpert Omni, unveiled by Cepheid in 2015 and dubbed as the world’s most portable molecular diagnostic system, was predicted to decentralize and increase access to TB diagnosis [113,114]. However, the commercialization plans for the Gene Xpert Omni were aborted, and Cepheid has received petitions to reinstate the plan to commercialize the diagnostic system [115,116]. The development of the Cepheid’s Gene Xpert systems was supported by the Foundation for Innovative New Diagnostics (FIND) and the National Institutes of Health (NIH), among other investors [117]. According to Gotham et al., FIND is currently evaluating the Gene Xpert Omni, and it is expected to be commercially available in 2022 [117]. Cost is still an issue, however, as the lowest cost of the GeneXpert instrument is USD 11,530 [118] and the per test cost averaged USD 21 [119].

An ideal diagnostic case for SARS-CoV-2/Flu A & B would be a test of ≤ USD 1 that can simultaneously detect and differentiate between SARS-CoV-2/Flu A & B RNA in 15 to 60 min with a sensitivity and specificity of >98%. This test would have ≤2 user steps, all reagents prepackaged within, be equipment-free (or operated by a simple, portable, and handheld device ≤USD 10), be made of environmentally friendly material, and disposable. Moreover, the device, test and its reagents would be stable at room temperature with a shelf-life of about a year. Finally, if a device is necessary beyond the disposable test itself, it would be battery or solar powered, and able to transmit results remotely or by USB connection to a mobile phone. While this ideal use case is for differentiating SARS-CoV-2 from Influenza A/B, a similar multiplexed and inexpensive test would help greatly with other infections, such as UTIs, blood infections, and diarrheal disease [120,121]. Cancer resistance genes identification, cardiovascular disease prognosis, cytokines profiling, and epigenetic modification profiling are other areas where multiplex detection of biomarkers will be invaluable [18,24,122,123,124].

Lateral flow assays meet the standards for affordability and accessibility, so improving their accuracy could be the answer. Molecular tests already have high accuracy, so a different approach would be adapting molecular tests into a REASSURED format and decreasing their cost/complexity. While there is currently no such diagnostic device, the rapid emergence of new technology, such as machine-learning-assisted diagnostics, CRISPR-based diagnostics and nanofluidic technology, places such ideals within reach with further research and innovation.

## Figures and Tables

**Table 1:** REASSURED scores of 9 clinically available multiplex diagnostics. The scoring was assigned on a 1 to 3 scale based on developed criteria (Supplementary Materials, Table S1). The total score was obtained by finding the average score across all elements of REASSURED and dividing by 3.

Test (Multiplex Capacity)	R	E	A	S	S	U	R	E	D	Score
Accula dock Flu A/Flu B Test (2)	3	3	1	2	2	3	3	1	3	78%
Visby Medical Sexual Health (3)	3	3	-	3	3	3	3	3	3	100%
Franklin three9 COVID-19 (27)	3	3	3	3	3	1	3	3	3	93%
Binax Now Influenza A & B with DIGIVAL (2)	3	3	1	1	2	3	3	3	3	81%
BD Veritor™ Flu A + B with analyzer (2)	3	3	2	1	3	3	3	3	3	89%
Sofia^®^ 2 Flu + SARS antigen FIA (3)	3	2	1	1	2	3	3	3	3	81%
Acucy influenza A and B (2)	3	3	2	1	3	3	3	3	3	89%
CardioChek PA Analyzer with CHOL + HDL + GLU Panel (3)	3	3	3	-	-	3	3	3	3	100%
CuroL7 (6)	3	3	3	-	-	3	3	3	3	100%

## Data Availability

Not applicable.

## Supplementary Materials

The following supporting information can be downloaded at: https://www.mdpi.com/article/10.3390/bios12020124/s1, Table S1: Scoring scheme for assessing diagnostics on the REASSURED criteria. The scoring ranges from 3 to 1, 3 being the highest score and 1 being the lowest score; Table S2: Scoring scheme of clinical diagnostics on the REASSURED criteria [125]. Averages were calculated from the scores of the individual elements of the REASSURED criteria. The Overall score was calculated by expressing the average score as a percentage of 3, the highest achievable average score (Reference [125] is cited in the supplementary information under Table S2).
