# Supplementary material for: REASSURED Multiplex Diagnostics: A Critical Review and Forecast
Source: Biosensors (Basel). 2022 Feb 16;12(2):124. doi: 10.3390/bios12020124 (PMC8869588; doi:10.3390/bios12020124)
Supplement: Supplementary file 1 [file biosensors-12-00124-s001.zip › biosensors-1561495-supplementary.pdf]

Supplementary Information

REASSURED Multiplex Diagnostics: A Critical Review and Forecast

Jonas A. Otoo and Travis S. Schlappi *

Keck Graduate Institute, Claremont, CA 91711, USA; jotoo18@students.kgi.edu
* Correspondence: tschlappi@kgi.edu

Table S1. Scoring scheme for assessing diagnostics on the REASSURED criteria. The scoring ranges from 3 to 1, 3 being the highest score and 1 being the lowest score.

	**High (3)**	**Medium (2)**	**Low (1)**
**R**	Test has a portable battery reader system or can be connected to mobile phone. The reader has real-time connectivity and can transmit results data	Test has a portable battery reader system which is does not have real-time connectivity or cannot transmit results.	Test has a reader system which is not battery operated.
**E**	Saliva, urine, stool, nasal swab, cheek swab, vaginal swab, nasopharyngeal swab, throat swab	Finger prick, sputum	Venous blood, serum
**A**	x < $5	$5 < x < $20	x > $20
**S**	x > 95%	90% < x < 95%	x < 90%
**S**	x > 95%	90% < x < 95%	x < 90%
**U**	x < 2 minutes, CLIA waived	2< x < 5 minutes, moderate complexity	x > 5 minutes, high complexity
**R**	x < 1 hr	1< x < 3 hrs	x > 3 hrs
**E**	Portable, handheld, disposable device or cartridge, battery or solar powered.	Portable, disposable device or cartridge, that can potentially be powered by a power pack.	Portable device or cartridge, potentially powered by power pack.
**D**	Reagents can be stored at room temp	Reagents are stable at room temp but for a few hours	Reagents require refrigeration

Table S2. Scoring scheme of clinical diagnostics on the REASSURED criteria. Averages were calculated from the scores of the individual elements of the REASSURED criteria. The Overall score was calculated by expressing the average score as a percentage of 3, the highest achievable average score.

Multiplex diagnostic	*Test type*	*CLIA*	**Cost of device*	*Cost of test*	*Test duration (min)*	*Hands-on time (min)*	*Sensit-ivity*	*Specific-ity*	*PPA*	*NPA*	*Temp*	*Sample*
Accula system flu A+B[73]	Nucleic Acid	Waived	$350	$63	30	1	94%	94%	-	-	RT	Nasal Swab
Visby Medical Sexual Health Click Test chlamydia, gonorrhoeae and trichomonas[75]	Nucleic Acid	Waived	-	-	28	1	98.80%	95.80%	97.40%	96.70%	RT	Vaginal swab
Franklin three9 Covid-19[76], [125]	Nucleic Acid	High	$9,950	$1.15	60	2	97.46%	98.51%	-	-	RT	NPS
Acucy influenza A+B[63]	Immunoassay	Waived	$1,454	$14.52	15	1	82.30%	96.00%	-	-	RT	NPS
Binaxnow influenza A+B with digival[61]	Immunoassay	Waived	$5,192	$28.68	15	1	-	-	81%	93.60%	RT	NPS
BD Veritor™ Flu A + B with analyzer[62]	Immunoassay	Waived	$411.55	$15.15	11	1	-	-	81.30%	97.60%	RT	NPS
Sofia 2 Flu + SARS antigen FIA[64]	Immunoassay	Waived	$2,080	$51.04	15	1	-	-	89%	94.60%	RT	NPS
CardioChek PA Analyzer[77]	Chemistry	Waived	$778	$10.53		1	-	-	-	-	RT	Finger pick
CuroL7 Blood profile test strips[78]	Chemistry	-	$289	$7.90	3	1	-	-	-	-	RT	Finger prick

*Cost of test column represents the average cost per test.
